# Methanol poisoning outbreak in Saudi Arabia: a case series

**DOI:** 10.1186/s13256-022-03600-7

**Published:** 2022-10-06

**Authors:** Rawan Eskandrani, Khalid Almulhim, Abdulla Altamimi, Abeer Alhaj, Shahd Alnasser, Laale Alawi, Eman Aldweikh, Khalid Alaufi, Bandr Mzahim

**Affiliations:** 1 Poison Control Center, Emergency Medicine Administration, King Fahad Medical City, Prince Abdulaziz Ibn Jalwi St, As Sulimaniyah, Riyadh, 12231 Saudi Arabia; 2 Emergency Medicine Department, Prince Mohammed Bin Abdulaziz Hospital, Riyadh, Saudi Arabia; 3 Emergency Medicine Administration, King Fahad Medical City, Riyadh, Saudi Arabia

**Keywords:** Methanol toxicity, Methanol poisoning outbreak, Saudi Arabia

## Abstract

**Background:**

Outbreaks of methanol poisoning have been described in the medical literature in different regions around the world. Even though in Saudi Arabia a few outbreaks of methanol poisoning have occurred, they remain undocumented. Herein, we describe several cases of methanol poisoning in Saudi Arabia with the goal of increasing awareness about the dangers of methanol poisoning among healthcare staff.

**Case presentation:**

Nine middle-aged Saudi patients (five men aged 24, 26, 27, 36, and 49 years and four females aged 19, 20, 24, and 25 years) were admitted to our emergency department after alcohol consumption. All patients presented with severe metabolic acidosis and some visual impairment. Treatment was initiated based on the clinical suspicion of methanol intoxication because of laboratory test limitations and time constraints. Patients showed improvement and favorable hospital outcomes after aggressive empirical treatment.

**Conclusions:**

Many social and cultural factors influence the lack of reporting of methanol poisoning cases in Saudi Arabia. We believe it is important to document these outbreaks to increase the knowledge among healthcare providers and promote public health awareness. A high index of suspicion and the development of local public health networks to monitor, survey, follow-up, and facilitate data exchange can help healthcare providers recognize and aggressively treat affected individuals. Early empiric and aggressive management can greatly decrease morbidity and mortality despite challenges and limited resources.

## Background

Methanol, also known as methyl alcohol, is a volatile, colorless, flammable fluid [1]. It is also known as wood alcohol because it is distilled from wood. Methanol is considered highly toxic and is usually only used for industrial purposes. However, it is sometimes used as an inexpensive and illegal counterfeit alternative to ethanol, especially in countries where ethanol consumption is restricted. Homemade alcoholic beverages are consumed as cheaper and more cost-effective underground ethanol substitutes [2, 3]. Recognizing methanol poisoning outbreaks is often challenging because of different religious, cultural, and societal reasons, and this can hinder early recognition and effective treatment [4–7]. Resource limitations can also contribute to the morbidity and mortality related to methanol ingestion [1, 5, 6]. Consuming ethanol simultaneously with methanol can have deleterious effects in treatment because it can significantly delay symptom presentation and, thus, diagnosis [4, 8, 9]. Reports of poisoning outbreaks due to methanol consumption are, unfortunately, frequently made, and these are likely to increase when alcohol consumption is restricted for cultural reasons or when alcohol is expensive because of high taxes. Therefore, healthcare providers should be vigilant, with a high index of suspicion for methanol poisoning cases because the morbidity and mortality depend on the interval between ingestion and initiation of therapy. Patient outcomes can be improved if methanol poisoning is recognized and treated aggressively before the methanol is metabolized by alcohol dehydrogenase into its toxic metabolite, formic acid [8]. Herein, we describe several cases of methanol poisoning, with the aim of raising awareness among healthcare staff about the dangers of methanol poisoning.

## Case presentation

The first case was a 49-year-old Saudi man who was found unconscious by his brother. He was brought to the emergency department (ED) by emergency medical services (EMS). On arrival, he showed decreased consciousness, hyperventilation, low-grade fever, and tachycardia. His family initially suspected that his condition was attributable to a drug overdose. He was intubated and mechanically ventilated because his Glasgow coma scale (GCS) score remained 9 despite efforts to stimulate and resuscitate him. His initial vital signs were as follows: blood pressure, 136/84 mmHg; heart rate, 145 beats/minute; respiratory rate, 56 breaths/minute; oxygen saturation, 98% in room air; and glucose level, 10 mmol/L. His physical examination was unremarkable apart from the presence of bilateral pinpoint pupils. After initial stabilization in the ED, he was admitted to the intensive care unit (ICU) because of low GCS and unstable vitals. He was maintained on mechanical ventilatory support and vasopressors because of hypotension. During the second day of his stay in the ICU, he started to show signs of worsening mental status and his computed tomography (CT) scan showed evidence of intracranial hemorrhage. His stay was complicated by ongoing evidence of sepsis and end organ damage represented by acute kidney impairment, worsening coagulopathy, rhabdomyolysis, and positive blood culture screen, with evidence of infective endocarditis on transesophageal echocardiography. This was managed by antibiotics and aggressive fluid and inotropic support. One week after admission, he showed no improvement in mental status and worsening neurological weakness. His repeated magnetic resonance imaging showed new middle cerebral artery infarction and the possibility of multiple infarcts. Attempts at weaning him from ventilatory support were unsuccessful and ended with placement of tracheostomy insertion. The patient was discharged on nasogastric and tracheostomy tubes in a stable condition but with poor functional state.

Our second patient was a 24-year-old man who was brought to our institution by EMS after a road traffic accident. Upon initial assessment, he was found to be intoxicated and agitated. His GCS score was 13. His initial vital signs were as follows: blood pressure, 114/61 mmHg; heart rate, 95 beats/minute; respiratory rate, 20 breaths/minute; oxygen saturation, 98% on room air; and glucose level, 5.7 mmol/L. His initial examination showed a red left eye but was otherwise unremarkable; furthermore, his extended focused assessment with sonography in trauma (eFAST) examination was negative. Apart from agitation, the findings of the patient’s physical examination were normal. The patient was hemodynamically stable upon assessment, with no signs of bleeding or other obvious injuries. He was acidotic with a pH of 7.25, and his HCO_3_ level was 10 mEq/L, as determined by analysis of the initial venous blood gas sample. Serum osmolality was 290 mOsm/kg. He received a fomepizole infusion; however, soon after sobering and admission, he discharged himself against medical advice.

The third patient was a 26-year-old man with no history of serious diseases, who presented to the ED complaining of blurry vision, vomiting, and hyperventilation that started the previous day after the consumption of an unknown amount of alcohol. He was alert and oriented to time, place, and person, with a GCS score of 15. His initial vital signs were as follows: blood pressure, 141/75 mmHg; heart rate, 92 beats/minute; respiratory rate, 20 breaths/minute; oxygen saturation, 100% in room air; and glucose level, 5.2 mmol/L. Analysis of his venous blood sample showed a pH of 6.9, and the HCO_3_ level was 5.7 mEq/L. The anion gap in his serum sample was 20 mEq/L, with a measured osmolality of 310 mOsm/kg (reference range, 275–295 mOsm/kg). His vision continued to diminish after admission. His management included fomepizole infusion, hemodialysis, thiamine infusion, and application of sodium bicarbonate boluses. Two days after admission, his laboratory markers normalized and he was discharged back to his normal condition, except for his vision derangement where he was referred to an ophthalmology specialized clinic where follow-up was lost.

Our fourth patient was a 25-year-old woman with a history of iron deficiency anemia who presented to the ED complaining of shortness of breath, dizziness, nausea, left flank pain, blurry vision, and palpitations. These symptoms had progressed over the last 3 days with alcohol consumption before she presented at our institution. The patient was conscious and oriented to time, place, and person. Her initial vital signs were as follows: blood pressure, 138/94 mmHg; heart rate, 104 beats/minute; respiratory rate, 22 breaths/minute; oxygen saturation, 98% in room air; and glucose level, 4.7 mmol/L. Her venous blood gas pH was 7.32 and HCO_3_ level was 15 mEq/L. The measured serum osmolality was 280 mOsm/kg and the anion gap was 15 mEq/L.

During admission, she received four doses of fomepizole infusion as per the institution poison control center protocol (0.9 g intravenous loading dose then 0.6 g q 12 hours). After completion of therapy, she was discharged home in her normal baseline condition.

The fifth patient was a 20-year-old woman who had been admitted and treated in another hospital for 1 week for acidosis and intoxication. She presented to our ED complaining of a 2-day history of bilateral complete vision loss. She had no other complaints. Her initial vital signs were as follows: blood pressure, 107/55 mmHg; heart rate, 54 beats/minute; respiratory rate, 20 breaths/minute; oxygen saturation, 99% in room air; and glucose level, 6 mmol/L. Her venous blood gas pH was 7.3 and HCO_3_ level was 14.9 mEq/L. The measured serum osmolality was 278 mOsm/kg, and the anion gap was 15 mEq/L. Upon admission, the patient received fomepizole infusion and was admitted for 3 days, during which time her condition improved, and she regained her vision. No hemodialysis was required. Patient was lost to follow-up after referral to ophthalmology.

Our sixth patient was a 27-year-old man who presented with complaints of vomiting and severe abdominal pain, restlessness, and blurry vision after alcohol consumption 2 days prior to his visit to our institution. His initial vital signs were as follows: blood pressure, 134/90 mmHg; heart rate, 84 beats/minute; respiratory rate, 20 breaths/minute; oxygen saturation, 98% in room air; and glucose level, 4.7 mmol/L. His venous blood gas showed a pH of 7.1, and the HCO_3_ level was 5 mEq/L. The measured serum osmolality was 296 mOsm/kg and the anion gap was 33 mEq/L. Methanol poisoning was strongly suspected, therefore, fomepizole, thiamine, folic acid, sodium bicarbonate infusion, and hemodialysis treatment were started. During his hospital stay, he received fomepizole as per the poison control center institution protocol (15 mg/kg intravenous loading dose of fomepizole followed by 10 mg/kg q 6 hours during his dialysis). He remained on continuous renal replacement therapy for 24 hours. He was discharged the next day after improvement and resolution of all his symptoms.

The seventh patient was a 36-year-old man who presented with complaints of fatigue, loss of appetite, and chest pain after consumption of alcohol 2 days prior to his visit to our institution. He had no visual symptoms and his GCS score was 15. The initial pH of his venous blood gas was 7.2, and the HCO_3_ level was 15 mEq/L. Serum osmolality was 277 mOsm/kg. He was administered fomepizole, folic acid, and sodium bicarbonate treatments and was soon discharged in good condition.

Our eighth patient was a 19-year-old woman with no history of serious diseases, who presented with acidosis, abdominal pain, vomiting, and blurred vision 2 days after the consumption of contaminated alcohol. The pH of her venous blood gas was 7.1, and the serum osmolality was 278 mOsm/kg. She was started on continuous renal replacement therapy upon admission, and given fomepizole, folinic acid, bicarbonate infusion until resolution of her symptoms was achieved. She was discharged back to her normal healthy baseline, with a referral to ophthalmology for follow-up.

The ninth patient was a 20-year-old woman who developed abdominal pain, nausea and vomiting, and blurred vision after attending a party. Analysis of her venous blood sample showed a pH of 7.34 and HCO_3_ level of 18 mEq/L. She was started on continuous renal replacement therapy upon admission and started on fomepizole infusion as per the poison control center institution protocol (15 mg/kg intravenous loading dose of fomepizole followed by 10 mg/kg q 6 hours during dialysis). She continued on treatment until resolution of her symptoms the next day. She was discharged back to her normal healthy baseline, with a referral to ophthalmology for follow-up.

## Discussion

Methanol poisoning is frequently encountered in vulnerable populations. Clandestine marketing of alcohol and the existence of local underground networks for distributing homemade alcohol result in the availability of cheaply and illegally produced counterfeit impure alcohol containing methanol, which could be ingested by unknowing consumers [1, 6].

### Methanol metabolism and formic acid toxicity

After its ingestion, methanol is metabolized by alcohol dehydrogenase into formaldehyde and then to formic acid, which produces toxic effects [1, 10]. Methanol toxicity has been linked directly to its slow metabolism and the accumulation of formic acid [11]. This has also been confirmed by Brent *et al.*, who identified a direct relationship between high serum formic acid concentrations and increased morbidity and mortality [12]. Two pathways have been identified for formic acid metabolism: the catalase-dependent pathway and the folate-dependent pathway, the latter of which is the major route of metabolism *in vivo* [8]. Hepatic concentrations of tetrahydrofolate have been shown to regulate the rate of formic acid oxidation [13]. The administration of folate has been observed to inhibit the increase in formic acid levels in the blood, indicating its metabolism to carbon dioxide [11]. The intermediate byproduct of methanol metabolism prior to formic acid formation is formaldehyde. While formaldehyde is potentially toxic, it is rapidly metabolized to formic acid and thus not detected in body fluids after methanol ingestion [9]. Methanol itself is not considered toxic, and its toxicity is related to its byproduct metabolites.

### Clinical features

The most commonly observed clinical features of methanol consumption are inebriation, central nervous system (CNS) depression, nonspecific gastrointestinal symptoms, metabolic acidosis, and visual disturbances [11]. The signs and symptoms are usually delayed and can appear up to 12–24 hours after ingestion. With higher concentrations of methanol, a half-life extension of up to 27 hours can be seen due to saturation of the elimination metabolic pathways [13]. The half-life can be extended further in cases of simultaneous methanol and ethanol ingestion [9].

### Optic toxicity

Undissociated formic acid specifically targets the optic disc and the retrolaminar section of the optic nerve. The toxicity induced by formic acid is greatly exacerbated by metabolic acidosis because it enables the increased diffusion of formic acid into the cells. Visual field defects, blurred vision, double vision, photophobia, and total vision loss are frequently encountered symptoms [11, 14].

In our region, sporadic incidents of methanol poisoning outbreaks have been reported over the years; however, the cases are under-reported. The reasons for this under-reporting are varied and include under-recognition of nonspecific symptoms leading to significant deleterious effects, incident occurrence at remote locations hindering timely access to professional help, low awareness of the direct link between counterfeit alcohol ingestion and the observed symptoms, cultural prohibition, social pressure, and a lack of a unified official entity for the reporting of such incidents. In one of the few studies on this topic conducted in Saudi Arabia, 50 consecutive patients were evaluated for ophthalmologic complications following methanol ingestion. In this study, it was unclear if patients were treated acutely or if they received timely therapy for methanol ingestion [15]. Most patients showed different optic disc abnormalities and variable degrees of vision loss. In another longitudinal cross-sectional study, 8 of 37 patients who did not have a subjective complaint of visual disturbance upon discharge from the hospital had abnormal findings in the retinal nerve fiber layer and visual evoked potential tests. In this study, the findings for 40% of all examined patients were consistent with long-term visual damage [16]. The targeted specificity of formic acid toward the optic nerve, retina, and basal ganglia has consistently been shown and confirmed in recent years [14–19].

### Neurotoxicity

Many patients with methanol poisoning have bilateral putamen necrosis, which is the most prominent CNS injury type. Diffuse bilateral white matter hypodensities, bilateral occipital necrosis, and bilateral intracerebral hemorrhage have also been noted in affected patients [20–22]. Damage to the basal ganglia, specifically in the putamen areas, is thought to be caused by the failure of the Na-K ATPase pump, inhibition of cytochrome oxidase, and local cellular edema [23]. It is important to note that bilateral necrosis of the putamen is not specific to methanol toxicity and can be seen in other conditions, such as familial neurodegenerative disorders and Wilson’s disease [9]. Lesions observed in the basal ganglia on imaging may not correlate with clinical outcomes and have been documented to not result in permanent dysfunction if the resolution of the putamen lesions occurs within 1 month of toxicity [24].

### Interpretation of serum methanol concentrations

The complicated association between serum methanol concentration and its clinical effects makes interpretation challenging. Individual variation, sample timing, and concomitant ethanol use are important factors to consider when assessing methanol ingestion. Furthermore, endogenous methanol production can lead to the presence of detectable methanol concentrations in some patients [25]. Concomitant ethanol use can reduce methanol toxicity and delay clinical manifestations [26]. This is because ethanol competitively inhibits methanol metabolism to its toxic compound, formic acid, by acting as the substrate for the alcohol dehydrogenase enzyme.

### Fomepizole as an antidote

The affinity of ethanol for alcohol dehydrogenase is 10-fold that of methanol [27]. In addition to ethanol, fomepizole has been found to be a potent inhibitor of alcohol dehydrogenase activity on methanol [28–30]. In the above-mentioned cases, the early use of fomepizole soon after methanol ingestion likely played a major role in the protection against and reduction of damage, and the better outcomes in some patients. Early administration of fomepizole has been shown to prevent metabolic acidosis and reduce the incidence of ocular toxicity in animals [31, 32]. The necessary fomepizole plasma concentration is approximately 0.8 µg/mL [32, 33]. Administration of a loading dose of 15 mg/kg body weight, followed by further intravenous bolus administration at doses of 10 mg/kg every 12 hours for four doses, followed by 15 mg/kg every 12 hours was sufficient to produce serum concentrations higher than 0.8 mg/L [12]. As indicated in a previously published case report, fomepizole infusion of 1–1.5 mg/kg/h during dialysis is sufficient to competitively inhibit methanol metabolism [34].

### Folinic acid

In all our cases of methanol poisoning, we empirically started folinic acid infusion due to the high safety margin and potential benefits associated with this treatment, although no human clinical trials have been conducted that can support the use of folinic acid in enhancing the metabolism of formic acid [35]. However, there is anecdotal support for the use of this treatment, along with supportive evidence from studies in animal models [35–37]. In the management of methanol toxicity, it is imperative to focus on supportive care to correct any electrolyte or metabolic disturbance and to prevent methanol metabolism. It has been postulated that the greater the efficiency of the folate pathway and the presence of folate derivatives, the greater the efficiency of the metabolic pathway and the enhanced elimination of formic acid [36, 38, 39]. Folic acid is converted into tetrahydrofolic acid derivatives, which are the primary bioactive forms of folate in the human body [40]. Folinic acid, the reduced form of folic acid, is preferred to folic acid in the case of methanol poisoning because it does not require any metabolic reduction. Folic acid is still considered an acceptable alternative if folinic acid is not available. The use of 1 mg/kg/body weight up to a total dose of 50 mg intravenously every 4–6 hours is suggested; however, the optimal dose of folinic acid remains to be established [38].

### Hemodialysis as an elimination method

The degree of morbidity and mortality in methanol poisoning have been linked to metabolic acidosis and not serum methanol concentration [41–43]. The therapeutic option of fomepizole administration alone versus hemodialysis with fomepizole administration is highly based on individual scenarios, clinical presentations, and hospital resources. Methanol has a prolonged elimination time. Hemodialysis is frequently recommended as a method for rapid methanol elimination when prolonged hospital stays and costs are impractical. Fomepizole administration can obviate the need for hemodialysis and requires less intensive monitoring [12, 44]. In the following cases, hemodialysis should be strongly considered: significant metabolic acidosis (pH < 7.25), visual disturbance, deterioration of vital signs despite the initiation of therapy, renal impairment, and serum methanol concentration of > 50 mg/dL [45–48]. During hemodialysis, increased infusion of the antidote, that is, ethanol or fomepizole, should be considered. Furthermore, antidote infusion should continue even after hemodialysis cessation, along with close monitoring for any rebound increase in serum methanol concentration [49].

## Conclusion and limitations

In our study, all presented cases occurred over a period of 2 weeks, possibly due to the prospective patients attending gatherings within the same time period. A common scenario leading to methanol poisoning was the attending of a social gathering and symptom development 2 days after alcohol consumption. Some presentations were delayed because patients feared legal consequences and deferred their visit to a healthcare facility until symptoms worsened and became unbearable, or until they feared permanent vision loss. Multiple other cases of methanol poising were encountered within the same timeframe across multiple healthcare facilities in the same city. However, reports of those cases were limited, as reporting is not mandatory and is left to the discretion of the treating physician. Approximately 25 affected individuals presented to healthcare facilities over that time period. It is highly plausible that most affected individuals had milder symptoms and did not visit a healthcare facility. It should be noted that none of the individuals could identify the source of the alcohol they consumed, and it is highly likely that they consumed counterfeit impure alcohol containing methanol. Unfortunately, this could not be proven.

One of the major limitations of our work was our inability to confirm methanol toxicity in blood samples because, despite its importance, it is not a routinely requested test. In all cases, empirical treatment was started based on patients’ medical history or a high clinical suspicion of methanol toxicity. The lack of awareness about the importance of requesting a concomitant ethanol level measurement as part of the management plan needs to be addressed.

This report is mainly meant to raise awareness among healthcare staff about the dangers of methanol toxicity in our region. The existence of clandestine markets and underground local networks for the distribution of homemade alcohol that do not have regulatory oversight is common. The development of local protocols for treatment and guidelines for laboratory measurement in an efficient and timely manner is an explicit need that should be addressed in the near future. Seeking medical care for the safe and effective management of methanol poisoning should be publicized to prevent unwanted morbidity and mortality. Ensuring proper follow-up after acute symptom presentation and ensuring access to psychological and addiction management clinics is also an important aspect to be considered.

## Data Availability

All data generated or analysed during this study are included in this published article.
